# A Pan* Plasmodium* lateral flow recombinase polymerase amplification assay for monitoring malaria parasites in vectors and human populations

**DOI:** 10.1038/s41598-024-71129-4

**Published:** 2024-08-30

**Authors:** Matthew Higgins, Mojca Kristan, Emma L. Collins, Louisa A. Messenger, Jamille G. Dombrowski, Leen N. Vanheer, Debbie Nolder, Christopher J. Drakeley, William Stone, Almahamoudou Mahamar, Teun Bousema, Michael Delves, Janvier Bandibabone, Sévérin N’Do, Chimanuka Bantuzeko, Bertin Zawadi, Thomas Walker, Colin J. Sutherland, Claudio R. F. Marinho, Mary M. Cameron, Taane G. Clark, Susana Campino

**Affiliations:** 1 Faculty of Infectious and Tropical Diseases, London School of Hygiene and Tropical Medicine (LSHTM), Keppel Street, London, WC1E 7HT UK; 2 Human Malaria Transmission Facility, LSHTM, Keppel Street, London, WC1E 7HT UK; 3 Environmental & Occupational Health, School of Public Health, University of Nevada, Las Vegas, USA; 4 Department of Parasitology, Institute of Biomedical Sciences, University of São Paulo, São Paulo, Brazil; 5 Malaria Reference Laboratory, UK Health Security Agency, LSHTM, London, WC1E 7HT UK; 6 Malaria Research & Training Center, Faculty of Medicine, Pharmacy and Dentistry, University of Science, Techniques and Technologies (USTT), Bamako, Mali; 7 Department of Medical Microbiology, Radboud University Nijmegen Medical Centre, Nijmegen, The Netherlands; 8 Laboratoire d’Entomologie Médicale Et Parasitologie, Centre de Recherche en Sciences Naturelles (CRSN/Lwiro), Sud‑Kivu, Democratic Republic of the Congo; 9 Médecins Sans Frontières (MSF) OCBA, Barcelona, Spain; 10 Institut de Recherche en Sciences de La Santé (IRSS), Bobo-Dioulasso, Burkina Faso; 11 Centre de Recherche en Sciences Naturelles de Lwiro, Sud-Kivu, Democratic Republic of the Congo; 12 Université Officielle de Bukavu (UOB), Bukavu, Democratic Republic of the Congo; 13 School of Life Sciences, University of Warwick, Coventry, UK; 14 Faculty of Epidemiology and Population Health, LSHTM, Keppel Street, London, WC1E 7HT UK

**Keywords:** Biological techniques, Molecular biology

## Abstract

Robust diagnostic tools and surveillance are crucial for malaria control and elimination efforts. Malaria caused by neglected *Plasmodium* parasites is often underestimated due to the lack of rapid diagnostic tools that can accurately detect these species. While nucleic-acid amplification technologies stand out as the most sensitive methods for detecting and confirming *Plasmodium* species, their implementation in resource-constrained settings poses significant challenges. Here, we present a Pan *Plasmodium* recombinase polymerase amplification lateral flow (RPA–LF) assay, capable of detecting all six human infecting *Plasmodium* species in low resource settings. The Pan *Plasmodium* RPA-LF assay successfully detected low density clinical infections with a preliminary limit of detection between 10–100 fg/µl for *P. falciparum.* When combined with crude nucleic acid extraction, the assay can serve as a point-of-need tool for molecular xenomonitoring. This utility was demonstrated by screening laboratory-reared *Anopheles stephensi* mosquitoes fed with *Plasmodium*-infected blood, as well as field samples of *An. funestus* s.l. and *An. gambiae* s.l. collected from central Africa. Overall, our proof-of-concept Pan *Plasmodium* diagnostic tool has the potential to be applied for clinical and xenomonitoring field surveillance, and after further evaluation, could become an essential tool to assist malaria control and elimination.

## Introduction

Malaria, resulting from infection with parasites belonging to the genus *Plasmodium*, remains a pressing global health issue, notably impacting nations situated in tropical and subtropical areas^1^. Although the scale-up of interventions has significantly reduced the malaria burden over the last two decades, an estimated 249 million cases and over half a million deaths still occur annually, with young children being the most affected^2^. In recent years many challenges, including, the emergence of drug and insecticide resistance, insufficient funding, and the effects of SARS-CoV-2 pandemic, have hindered malaria control progress, leading to a reversal in global elimination outcomes^3–6^. To overcome such challenges the development of new technologies is vital to aid and enhance existing control efforts.

A critical component of any malaria control strategy is pathogen monitoring and surveillance. This information is essential for guiding intervention efforts, identifying high-risk geographical regions or populations, providing outbreak warnings, and monitoring malaria transmission patterns^7^. Malaria transmission has commonly been determined by tracking the prevalence of *Plasmodium* parasites in the human population at a given time point (parasite rate; PR), using immunochromatographic-based rapid diagnostic tests (RDTs) or microscopy, which remains the gold-standard diagnostic method for malaria^8–10^. Xenomonitoring is a complementary approach, establishing the proportion of vectors harbouring a pathogen of interest, within a geographical region. Several techniques can be used for malaria xenomonitoring. One approach involves mosquito dissection by trained entomologists to confirm the presence of *Plasmodium* sporozoites, thereby assessing the mosquito's ability to transmit the disease. Additionally, molecular techniques such as polymerase chain reaction (PCR) can detect the presence of *Plasmodium* DNA in mosquitoes, regardless of the parasite stage. However, PCR alone does not indicate the mosquito's capacity to transmit malaria^11,12^. When combined with other metrics, such as the entomological inoculation rate (EIR)—the average number of infectious bites received per person—xenomonitoring can help assess the impact of intervention deployment on malaria infection dynamics and provide a comprehensive picture of ongoing disease transmission.

The accuracy and precision of both PR and EIR estimates depend on the efficacy of the methods used, particularly the ability to detect low-density infections in both human and *Anopheles* spp populations, which still contribute to malaria transmission. Nucleic-acid amplification technologies (NAATs), such as quantitative PCR (qPCR) and loop-mediated isothermal amplification (LAMP), are the most sensitive techniques for detecting *Plasmodium* spp, outperforming other diagnostic methods like RDTs and microscopy^13–15^. However, NAATs typically require laboratory access, expensive equipment, and trained personnel, which are challenging to obtain in resource-limited settings.

To overcome these challenges, we sought to develop a Pan *Plasmodium* recombinase polymerase amplification (RPA)-based lateral flow (LF) assay (“Pan Plasmodium RPA-LF" assay). This assay aims to match the sensitivity of other NAATs while being suitable for use in low-resource field settings. It is designed to detect any of the six human malaria species (*P. falciparum, P. vivax, P. malariae, P. knowlesi, P. ovale curtisi,* and *P. ovale wallikeri)*. The assay operates at a single temperature between 37–42 °C, which allows for the possibility of being powered by human body heat. The lower incubation temperature of our assay, compared to other isothermal NAATs such as LAMP (65 °C), is achieved using recombinase proteins (UvsX, UvsY). These proteins facilitate strand exchange between ssDNA primer oligos and dsDNA targets, eliminating the need for the denaturation and annealing steps typical in a standard PCR cycle^16^. Additionally, the assay reagents can be lyophilized, which improves shelf-life during storage and transportation, and removes the dependence on a cold chain, which can be unreliable in resource-limited settings.

In this work, we evaluate the Pan Plasmodium RPA-LF assay using a combination of clinical samples and both laboratory-reared and field-collected *Anopheles* spp mosquitoes. This provides a proof-of-concept assessment for the assay's use as a multifaceted surveillance tool. Moreover, this study demonstrates the viability of a crude nucleic acid extraction approach in tandem with the Pan Plasmodium RPA-LF assay, enhancing its applicability for point-of-need molecular xenomonitoring in resource-limited settings.

## Results

### Assay design & in silico screening

The Pan Plasmodium RPA-LF assay was specifically designed for this study following established guidelines to detect all six human-pathogenic species of malaria: *P. falciparum, P. vivax, P. knowlesi, P. malariae, P. ovale curtisi,* and *P. ovale wallikeri*^17^. It targets the mitochondrial rRNA encoding region of *Plasmodium* spp, which encompasses a total of 5 genes (Pf3D7 Orthologs: *PF3D7_MIT03800, PF3D7_MIT03900, PF3D7_MIT04000, PF3D7_MIT04100, PF3D7_MIT04200*; sourced from PlasmoDB). Inclusivity of the assay was assessed in silico against a dataset of 890 publicly available mitochondrial sequences from all human-infecting *Plasmodium* species (see Supplementary Information). All oligonucleotide binding sites were conserved across 99.4% (885/890) of sequences screened (Supplementary Table 1). In addition, the specificity of the Pan Plasmodium RPA-LF assay was assessed in silico against the nt database using the NCBI Blastn tool. No alternative binding sites were identified in the human host (*Homo sapiens* (TaxID: 9606)) or vector (*Anopheles* spp (TaxID: 7164)), with 91.8% of identified hits originating from sequences classified under *Plasmodiidae* (see Supplementary Table 2).

### Assay inclusivity and limit of detection (LoD)

**Fig. 1:**
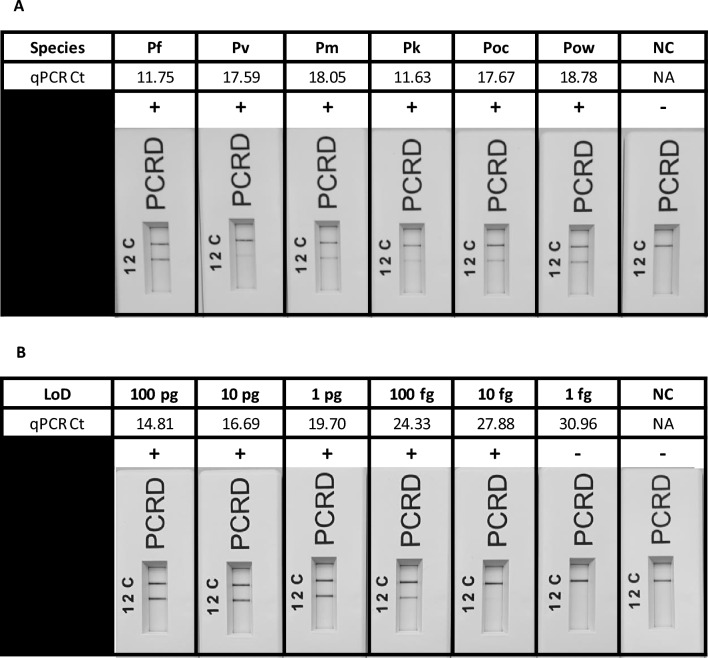
(**A**) Inclusivity assessment for the Pan Plasmodium RPA-LF assay across all 6 human infecting *Plasmodium* species. *P. falciparum* (Pf), *P. vivax* (Pv), P*. malariae* (Pm), *P. knowlesi* (Pk), *P. ovale curtisi* (Poc), *P. ovale wallikeri* (Pow). (**B**) Limit of detection (LoD) assessment for the Pan Plasmodium RPA-LF assay for *P. falciparum*. Lateral flow cassettes have been cropped to remove the loading port. Lane C (C) represents the flow-check line and Lane 2 (2) detects the FAM/Biotin labelled amplicons generated via the Pan Plasmodium RPA-LF assay.

Following in silico assessment, the Pan Plasmodium RPA-LF assay was first validated across all six human infecting *Plasmodium* species. For *P. falciparum (Dd2) and P. knowlesi (A1-H.1),* DNA extracted from cultured strains was utilised. For the other unculturable *Plasmodium* species (e.g., *P. vivax*, *P. malariae*, *P. ovale curtisi* and *P. ovale wallikeri*)*,* DNA was extracted from clinical isolates, kindly provided by the UK Health Security Agency—Malaria Reference Laboratory (UKHSA-MRL, LSHTM), was used. All species were successfully detected (Fig. 1A, Supplementary Fig. 1), and the results were compared with qPCR performed in tandem (see Methods section). Cultured *Plasmodium* isolates had the lowest qPCR Ct values, with *P. falciparum* at 11.75 and *P. knowlesi* at 11.63, compared to clinical isolates which had a Ct range of 17.59–18.78, reflecting the high parasitaemia achievable in culture. Following inclusivity screening, the sensitivity of the Pan Plasmodium RPA-LF assay was assessed against *P. falciparum.* The use of parasite cultures ensured that only parasite DNA was present, unlike clinical isolates which contain a mix of parasite and human DNA from white blood cells. This facilitated accurate fluorescent-based quantification of *P. falciparum* using the Qubit high sensitivity DNA detection assay. Initial screening established the limit of detection (LoD) for the Pan Plasmodium RPA-LF assay against *P. falciparum* to be between 100 fg DNA/µl (10/10) and 10 fg DNA/µl (9/10) (Fig. 1B, Supplementary Fig. 2).

### Clinical application

**Fig. 2:**
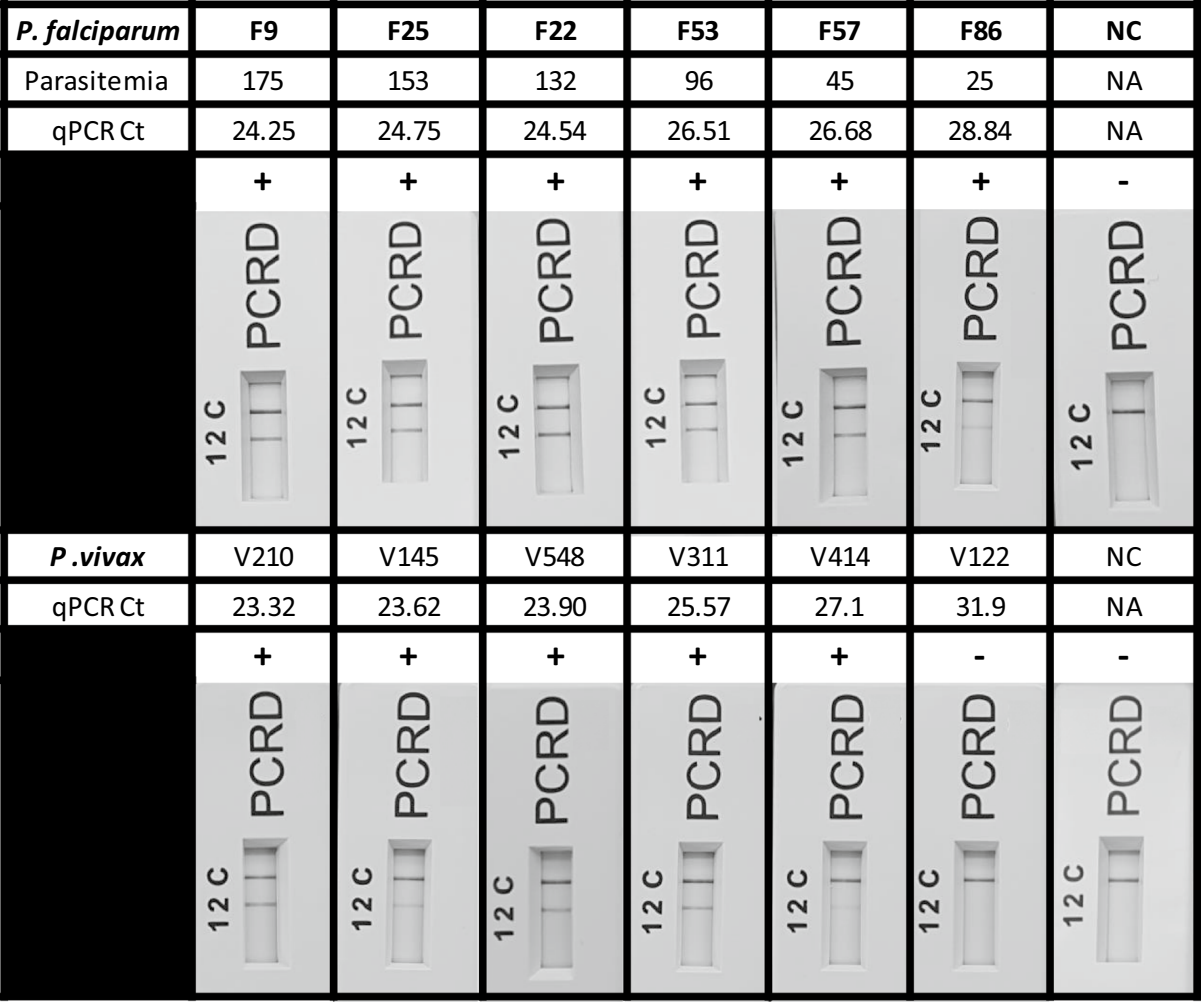
A subset of the *P. falciparum* and *P. vivax* clinical isolates screened using the Pan Plasmodium RPA-LF assay, including the negative *P. vivax* isolates (V122). Lateral flow cassettes have been cropped to remove the loading port. Lane C (C) represents the flow-check line and Lane 2 (2) detects the FAM/Biotin labelled amplicons generated via the Pan Plasmodium RPA-LF assay.

To assess the Pan Plasmodium RPA-LF assay for potential clinical use, 46 isolates (24 *P. falciparum*, 12 *P. vivax*, 5 *P. malariae*, and 5 *P. ovale* spp.), previously diagnosed via microscopy, were screened. The assay successfully detected all *P. falciparum* (24/24) and most *P. vivax* (11/12) infections (Fig. 2, Supplementary Tables 3 and 4). Among the *P. falciparum* isolates, fourteen had parasitaemia below 200 parasites/µl (0.004%), confirmed via microscopy and classified as low-density infections according to the WHO Malaria RDT Performance report^18,19^ (Supplementary Fig. 3).

Tandem qPCR screening of the same isolates indicated that the failed detection of a single *P. vivax* isolate was likely due to its concentration being below the LoD of the Pan Plasmodium RPA-LF assay, with a Ct > 31, indicative of a *Plasmodium* spp. concentration less than 1 fg/µl as established in Fig. 1 (Supplementary Table 4). Additionally, one RPA-LF positive *P. vivax* isolate tested negative via tandem qPCR screening.

Comparatively, the assay successfully detected all *P. malariae* (5/5) and *P. ovale* spp. (5/5) clinical isolates provided by the UKHSA-MRL (Supplementary Fig. 4, Supplementary Table 5). Across all Pan Plasmodium RPA-LF positive clinical isolates, the Ct values obtained via tandem qPCR were consistent with prior inclusivity screening. The highest Ct values for each species were *P. falciparum* (28.84), *P. vivax* (27.10), *P. malariae* (19.56), and *P. ovale* spp. (21.46).

### Xenomonitoring Proof-of-Concept

**Fig. 3:**
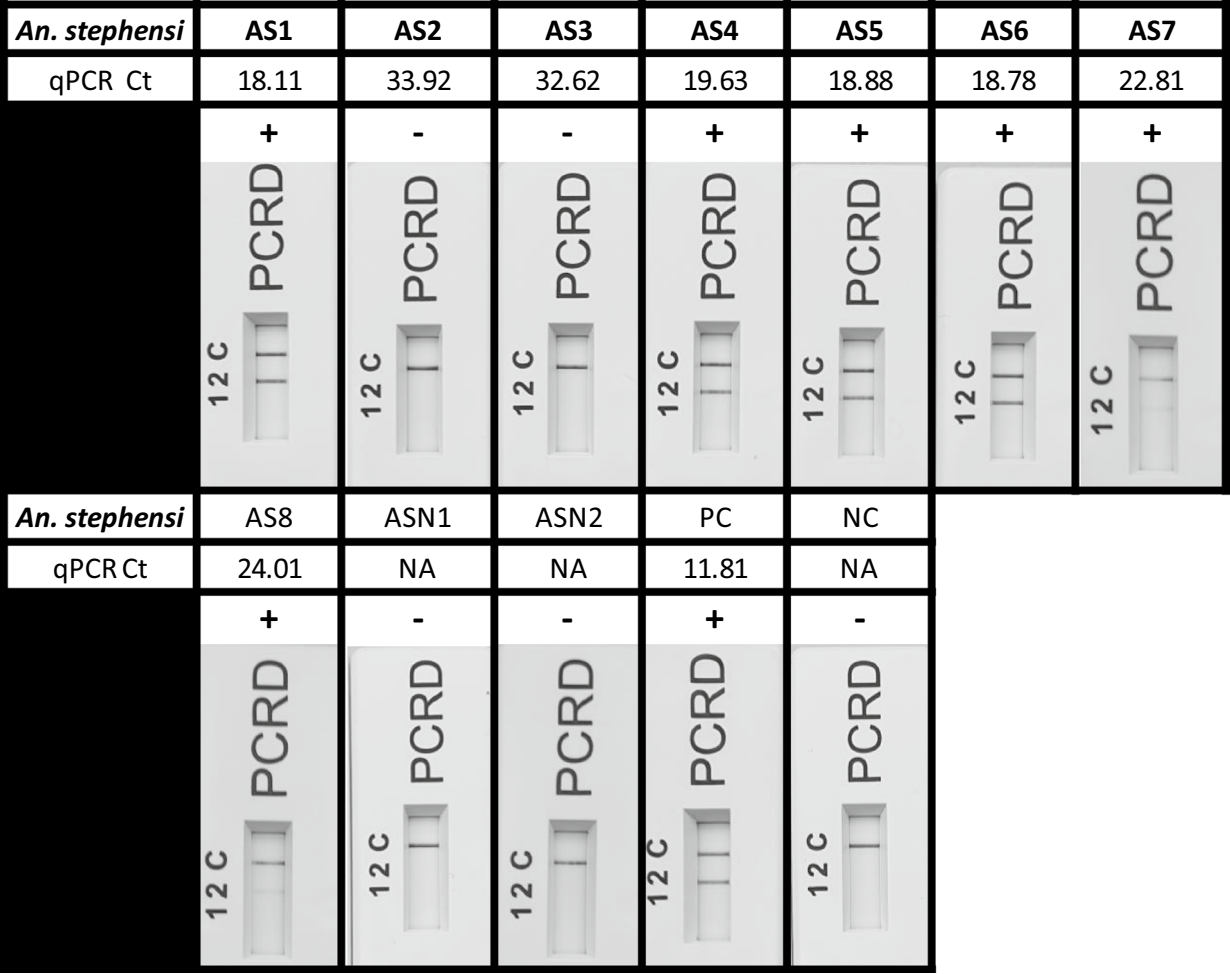
Screening of eight infected-blood-fed mosquitoes (AS1-8) and two uninfected *An. stephensi* mosquitoes (ASN1, ANS2), provided by the Human Malaria Transmission facility. The positive control (PC) and negative control (NC) consisted of *P. falciparum* and water, respectively. Lateral flow cassettes have been cropped to remove the loading port. Lane C (C) represents the flow-check line and Lane 2 (2) detects the FAM/Biotin labelled amplicons generated via the Pan Plasmodium RPA-LF assay.

To evaluate the assay’s potential for xenomonitoring applications, eight infected blood-fed and two uninfected *Anopheles stephensi* mosquitoes were provided by the Human Malaria Transmission Facility at LSHTM. Following DNA extraction using the Qiagen DNeasy Blood and Tissue Kit, all 10 vectors were screened with the Pan Plasmodium RPA-LF assay (Fig. 3). Six out of the eight infected blood-fed *An. stephensi* mosquitoes tested positive with the Pan Plasmodium RPA-LF assay. The two remaining infected mosquitoes (AS2, AS3) had parasite densities below the assay’s limit of detection, as indicated by qPCR Ct values of 33.92 and 32.62, respectively, as established in Fig. 1**.** Both uninfected *An. stephen*si mosquitoes were negative in both RPA and qPCR assays.

Subsequently, the Pan Plasmodium RPA-LF assay was assessed on 29 *Anopheles* spp. field samples, a mixture of *An. funestus* s.l. and *An. gambiae* s.l., collected between 2018 and 2019 from the Democratic Republic of the Congo (DRC). Tandem qPCR screening identified four out of the 29 field samples as positive (Ct value range: 25.45–30.31), consistent with previous molecular assessments conducted upon sample collection (Supplementary Table 6). However, the Pan Plasmodium RPA-LF assay successfully detected only two of these samples (Ct: 25.45, 27.23); the other two samples had Ct values (27.73, 30.31) below the assay's limit of detection. Among the two RPA-LF positive samples, only one had evidence of a recent blood meal, indicated by the presence of blood in the abdomen. The remaining 24 samples were negative by both qPCR and the Pan Plasmodium RPA-LF assay, aligning with prior molecular assessments (Supplementary Table 6).

### Crude nucleic acid extraction

Most malaria surveillance methods in molecular xenomonitoring typically involve transporting collected samples to a central facility for sorting, morphological identification, pooling, and processing—often via dissection or nucleic acid extraction—before screening^20^. To enable the use of the Pan Plasmodium RPA-LF assay directly at the point of collection, we have developed a room temperature (22 °C), polyethylene glycol (PEG)-enhanced chemical crude extraction method. This approach eliminates the need for specialised equipment and involves an alkaline lysis buffer based on potassium hydroxide, followed by neutralisation with a tris–acetate buffer (details in the Methods section)^21^. Following crude extraction, the sample can be directly introduced into the Pan Plasmodium RPA-LF assay.

**Fig. 4:**
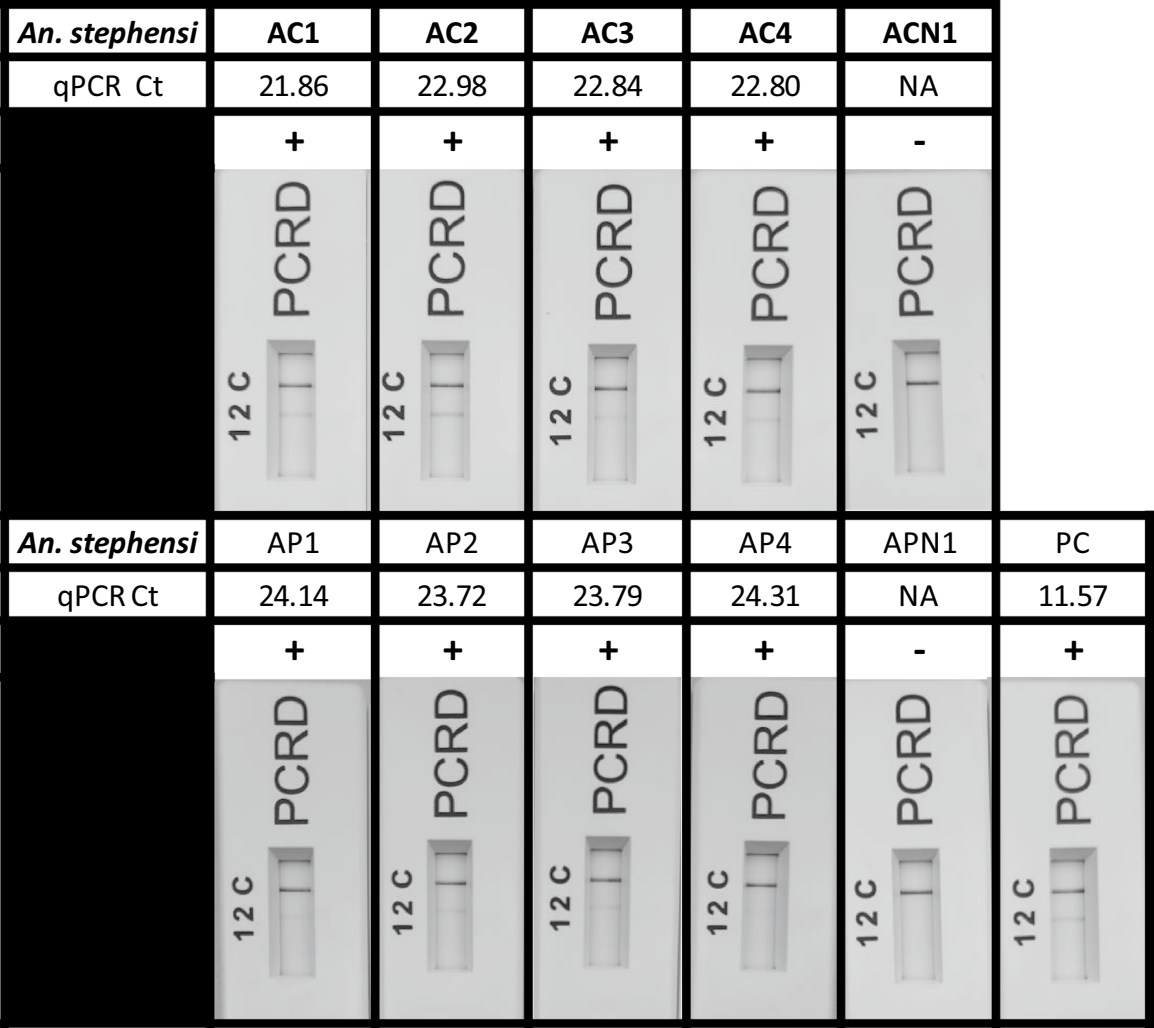
A subset of single (AC) and pools (AP) of infected-blood-fed *Anopheles* spp mosquitoes screening using the Pan Plasmodium RPA-LF assay following crude extraction. Lateral flow cassettes have been cropped to remove the loading port. Lane C (C) represents the flow-check line and Lane 2 (2) detects the FAM/Biotin labelled amplicons generated via the Pan Plasmodium RPA-LF assay.

We initially validated this extraction method using eight individual *An. stephensi* mosquitoes, each recently fed on infected blood and provided by the Human Malaria Transmission Facility at LSHTM. To ensure complete submersion, we employed 75 µl of lysis buffer and 25 µl of neutralization buffer per mosquito. Following extraction, all eight mosquitoes tested positive for infection upon screening (Fig. 4, Supplementary Table 7). Subsequently, we created ten pools of mosquitoes: eight pools contained one infected-blood-fed *An. stephensi* each, along with four unfed *An. stephensi*, while two negative control pools consisted of five unfed mosquitoes each. Pools containing infected-blood-fed vectors consistently tested positive in the Pan Plasmodium RPA-LF assay following crude nucleic acid extraction using 150 µl of lysis buffer and 50 µl of neutralisation buffer (Fig. 4, Supplementary Table 7).

## Discussion

In this study, we present a comprehensive Pan *Plasmodium* RPA-LF-based assay for disease surveillance, capable of detecting all six human-infecting *Plasmodium* species from clinical and cultured isolates. Our findings expand on previous applications of RPA-LF technology in malaria diagnostics, including the development of species-specific assays detecting four human-infecting species^22–24^. While *P. falciparum* bears the highest disease burden, monitoring all human-infecting species is critical for effective disease surveillance and tailored interventions. In silico analysis confirmed the assay's inclusivity across human-infecting *Plasmodium* species, essential for optimal performance as primer-template mismatches can otherwise lead to false negatives^25^. Subsequent screening of clinical isolates for *P. falciparum, P. vivax, P. malariae*, and *P. ovale* species demonstrated a detection rate of 97.8% (45/46), surpassing the WHO's 97% minimum sensitivity threshold for point-of-need malaria diagnostics^26^. However, further validation is needed through expanded screening of clinical isolates, including *P. knowlesi* and negative infections, and standardisation of DNA extraction methodologies across all samples. Additionally, assessing the assay's field applicability and the feasibility of using crude-extraction methods for processing human blood samples directly are essential.

Our study successfully detected low-density *P. falciparum* infections below the WHO's 200 parasites per µl 'low parasite density' threshold, as reported in their latest Malaria RDT Performance report^19,27^. The established limit of detection (LoD) for *P. falciparum* of 10–100 fg is comparable to other RPA-based pathogen assays^28–30^, aligning with the sensitivity of other *Plasmodium-*detecting NAATs such as mini-dbPCR-NALFIA and *P. knowlesi* RPA-LF, both with a sensitivity of 10 parasites/µl. Early detection of malaria is crucial for improving clinical outcomes^31,32^, highlighting the importance of achieving high sensitivity in our assay. For broader implementation by control programs, reducing the cost of the RPA-LF assay—currently £6.80 per reaction (TwistAmp® Basic 1 × Reaction: £3.87 and Abingdon PCRD LF Cassette £2.93 as of June 2024)—to align with other solutions is imperative. Furthermore, similar to existing Pan Malaria RDTs, positive results would require further species-specific screening.

The presence of *Plasmodium* spp. parasites was also evaluated across three *Anopheles* spp. Vectors—*An. stephensi, An. funestus* s.l., and *An. gambiae* s.l.—affirming the assay's effectiveness as a tool for molecular xenomonitoring surveillance of malaria. Testing reared *Anopheles* mosquitoes that had recently fed on infected blood demonstrated the capability to detect *Plasmodium* spp. before the establishment of infection in the mosquito. To our knowledge, this is the first study to demonstrate the detection of *Plasmodium* spp. using RPA-LF technology in both host and vector isolates. However, further enhancements are needed to align assay sensitivity with current vector screening practices, particularly evident in field samples collected from the DRC. This approach will allow for the detection of *Plasmodium* infections in vectors after blood meal digestion, regardless of the stage in the sporogonic cycle. Improvements may involve optimising the ratio of reaction mixture loaded onto the lateral flow cassette and increasing the concentration of recombinase proteins that aid in template priming and amplification. Such enhancements would eliminate the need to differentiate between blood-fed and unfed vectors during collection, prior to pooling and screening. This molecular approach is less labour-intensive than traditional mosquito dissections aimed at detecting sporozoites. Additionally, screening prior to blood meal digestion creates the opportunity to detect a wide range of parasites in hematophagous insects, regardless of their status as vectors. This approach could provide insights into the prevalence of *Plasmodium* infection within a given collection area.

The ability to operate RPA-based assays using human body heat and incorporate a lateral flow cassette for result detection reduces reliance on specialised equipment compared to other NAATs like LAMP and qPCR. Further validation work is needed to demonstrate the feasibility of using the RPA-LF assay in equipment-free settings with human body heat for incubation. Our presented Pan Plasmodium RPA-LF assay opens pathways for non-specialist use, akin to existing malaria RDTs or lateral flow-based diagnostics for SARS-CoV-2. When combined with crude nucleic acid extraction, this assay offers a point-of-need alternative for disease surveillance programs in resource-limited settings where qPCR facilities are unavailable. Consequently, it eliminates the need to transport and process samples at centralised laboratories, which is standard practice in most surveillance efforts. For instance, the assay could be integrated into ongoing field trapping and morphological speciation efforts to monitor the spread of the invasive *An. stephensi* species across Africa—a major concern highlighted by the WHO for malaria control. This approach can assess how the distribution of infected vectors influences malaria transmission and guide appropriate intervention strategies^33^. However, similar to other NAAT-based methods for vector incrimination, the methodology's utility should be considered alongside its potential to overestimate infection rates, as it detects any *Plasmodium* DNA present, not exclusively from infected mosquitoes harbouring sporozoites^11^.

In summary, we have demonstrated the versatile surveillance capabilities of the Pan Plasmodium RPA-LF assay, which, when paired with crude DNA extraction, has the potential to become a pivotal tool in malaria surveillance programs in field settings with further refinement.

## Methods

### Cultured and neglected plasmodium spp.

*Plasmodium falciparum (Dd2) and P. knowlesi (A1-H.1)* DNA was extracted from parasite cultures, kindly provided by Robert Moon (LSHTM), utilising the DNeasy Blood & Tissue Kit (Qiagen)*. P. malariae*, *P. ovale curtisi* and *P. ovale wallikeri* DNA samples were extracted from blood samples from infected travellers returning to the UK, who were diagnosed with malaria between 2019 and 2020, as confirmed by the UK Health Security Agency-Malaria Reference Laboratory at the LSHTM. Speciation of infections was performed by nested PCR and qPCR as outlined previously^34^. The UK National Research Ethics Service (Ref: 18/LO/0738) and LSHTM Research Ethics Committee (Ref: 14,710) provided approval for the project.

### *Plasmodium falciparum*

A total of 24 whole blood samples were selected from *P. falciparum* gametocyte carriers, aged between 5–50 years, recruited into one of the previously published clinical trials in Ouélessébougou, Mali^18,35^. Permission to conduct this study was obtained from the LSHTM Research Ethics Committee (Ref: 17,507) and the University of Sciences Techniques and Technologies of Bamako Ethical Committee (Ref: 2019/67/CE/FMPOS and 2020/96/CE/FMPOS/FAPH) and performed in accordance with relevant guidelines and regulations. The trial was registered on ClinicalTrials.gov (NCT04049916). Written informed consent was obtained from all subjects and/or their legal guardians prior to sample collection. For minor participants, informed consent for study participation was obtained from their parent and/or legal guardian. Species identification was carried out by microscopy by trained microscopists at the Malaria Research and Training Centre of the University of Bamako (Bamako, Mali). DNA was extracted from 83.3 μl whole blood using a MagNAPure LC automated extractor (Total Nucleic Acid Isolation Kit High Performance; Roche Applied Science, Indianapolis, IN, USA)^18^.

### *Plasmodium vivax*

A total of 12 whole blood samples were selected from *P. vivax* carriers, aged between 13–30 years, recruited as part of a cohort study of pregnant women, performed in the State of Acre, Brazil, between 2013–2015^36^. Permission to conduct this study was obtained from the Ethics Committee from the University of São Paulo-Brazil (CEP/USP) and the National Commission for Research Ethics (CONEP) (Plataforma Brasil, CAAE nº 32,707,720.0.0000.5467), and performed in accordance with relevant guidelines and regulations. Written informed consent was obtained from all subjects and/or their legal guardians prior to sample collection. Species identification was first performed by microscopy by the endemic surveillance team in the State of Acre and then confirmed by molecular diagnosis using the real-time PCR technique^37^. DNA was extracted from 200 μl whole blood using QIAamp DNA blood mini kit (Qiagen).

### Anopheles stephensi—human malaria transmission facility

A cohort of *An. stephensi* (SD500 strain) were fed with a NF54 *P. falciparum* asexual laboratory strain culture at approximately 1% parasitaemia, by the LSHTM Human Malaria Transmission facility team. A total of 24 blood fed mosquitoes were randomly selected 4 h post feed, in addition to 6 unfed mosquitos for further pooling and screening.

### Field *Anopheles spp*

A total of 29 *Anopheles* spp, field isolates were collected in 2018–2019, using indoor Centers for Disease Control and Prevention light traps in two sites in Sud-Kivu province (Tchonka and Tushunguti) and one site in Haut-Uélé province (Kibali), DRC. Following collection, vectors were speciated (*An. funestus* s.l. or *An. gambiae* s.l.) by morphology and classified as either blood-fed or unfed, by a trained entomologist. Genomic DNA was extracted from individual mosquitoes using a Qiagen DNeasy blood and tissue kit. The study protocol in Tushunguti and Tchonka was approved by the National Ethics Committee of Health in the DRC (Ref: CNES001/DPSK/111PM/2017). For the Kibali site, the protocol was approved by the scientific ethics committee of the Centre de Recherche en Sciences Naturelles of the Université Officielle de Bukavu. Permission to conduct this study was obtained from the LSHTM Research Ethics Committee (Ref: 25,808).

### Crude nucleic acid extraction

A crude nucleic acid extraction protocol suitable for RPA was developed consisting of a lysis buffer (100 mM Potassium Hydroxide, 25% Polyethylene Glycol) and neutralisation buffer (1 M Trizma base, 0.75 M Acetic acid) which are added in a 3:1 ratio. First 75 µl / 150 µl of lysis buffer was added to individual / pools of mosquitoes, followed by mechanical lysis using a pestle and a Kimble® Pellet Pestle® Cordless Motor (DWK Life Sciences, Germany) and incubated at room temperature (22˚C) for 30 min. Following incubation, 25 µl/50 µl of neutralisation buffer was added to rebalance the pH and stop further alkaline based lysis.

### Recombinase polymerase amplification (RPA)

TwistAmp Basic kits were supplied by TwistDx Ltd., Cambridge, United Kingdom. PCRD lateral flow cassettes were supplied by Abingdon Health Ltd, York, United Kingdom. A 10 × Primer–Probe master mix was created, (4 µM standard primer: GAGTCGATCAGGAAGGTTTCATCCTTAAAT, 4 µM biotinylated primer: /5Biosg/GTCTCTATGCCTTGAATGGAGCACTGGATTGG, 2 µM Nfo Probe: FAM/GTTAAGGTGCTCAGGGTCTTACCGTCGGGCCGTAT/idSp/ATTCCACATA/3SpC3/. Standard and modified oligonucleotides were supplied by Integrated DNA Technologies with the appropriate modifications, FAM (Fluorescein amidites), idSp (Int 1’,2’-Dideoxyribose), and blocking ground (3SpC3). Each reaction consisted of the TwistAmp Basic Freeze-dried enzyme pellet, rehydrated with 29.5 µl of Rehydration Buffer, 8 µl of water, 5 µl of 10 × Primer–Probe master mix and subsequently 3 µl of Endonuclease IV, provided by New England Biolabs LTD. To each reaction, 2 µl of sample was added, followed by 2.5 µl of Magnesium Acetate provide by TwistDX to initiate the reaction; the tube was mixed by inversion and brief centrifugation. Each reaction was run for 45 min at 39 ˚C. Upon reaction completion, 10 µl of reaction mix was added to 80 µl PCRD Buffer and mix via inversion. 75 µl of the mixed solution was then applied to the PCRD lateral flow cassettes. The presence of any bands was recorded after 15 min, and lateral flow cassettes were imaged using a Samsung Galaxy A42 5G smartphone camera.

### Quantitative real time PCR

Quantitative PCR was performed using the Kapa Probe Fast (ROCHE, Basel, Switzerland). Each reaction consisted of 10 µl KAPA PROBE FAST qPCR Master Mix, 6.6 µl DNase free water 0.4 50 × ROX Low, 1 µl of Prime-Probe Mix and 2 µl of sample, unless specified. Oligos for the *Pan*-*Plasmodium* qPCR assay designed for this study (Forward Primer: 5’-TYACGAGTCGATCAGGAAGG, Reverse Primer: 5’-TTCCCCATTGTCGCTAGTGT, Probe: 5’-/56-FAM/AAGGTGCTC/ZEN/AGGGTCTTAC C/3IABkFQ/ were ordered from Integrated DNA Technologies (Coralville, Iowa, United States). All qPCR reactions were performed using Stratagene Mx3005P (Aligent Technologies). Cycling conditions consisted of 3 min at 95˚C enzyme activation step, followed by 40 cycles of 15 s denaturation step at 95˚C and 45 s 60˚C annealing and extension step at the end of which fluorescence was recorded.

### Supplementary Information

Supplementary Information.

## Data Availability

A list of NCBI IDs for sequences used as part of the *in-silico* analysis can be found at GitHub (https://github.com/MatthewHiggins2017/PanPlasmodiumRPALFManuscript).
