# Supplementary material for: A Pan Plasmodium lateral flow recombinase polymerase amplification assay for monitoring malaria parasites in vectors and human populations
Source: Sci Rep. 2024 Aug 30;14:20165. doi: 10.1038/s41598-024-71129-4 (PMC11364753; doi:10.1038/s41598-024-71129-4)
Supplement: Supplementary file 1 — Supplementary Information. [file 41598_2024_71129_MOESM1_ESM.docx]

**A Pan Plasmodium lateral flow recombinase polymerase amplification assay for monitoring malaria parasites in vectors and human populations Supplementary Information**

***In-silico* Inclusivity Check**

Searching NCBI Nucleotide database using terms (txid5820 [Organism:exp] AND Mitochondrion), 1124 sequences were identified and downloaded, in addition to two *P. ovale spp* mitochondrial genomes (Poc221_MIT, Pow222_MIT: <https://github.com/MatthewHiggins2017/HigginsMPovaleReferences>). Sequences then underwent a quality control (QC) check, establishing minimum and maximum length thresholds as 5,800 and 6,200 respectively, excluding unknown (N) nucleotides. In addition, only human infecting *Plasmodium* sequences were included, and necessary sequences were reverse complemented to ensure all were the same sense. Following QC, 890 sequences were subsequently aligned using MAFFT (v7.310). Sequence IDs for the 890 sequences used can be found at (<https://github.com/MatthewHiggins2017/PanPlasmodiumRPALFManuscript>). Post alignment, BioPython was used to assess the conservation of each Pan-*Plasmodium* RPA-LF assay oligo binding site in each sequence respectively. Where more than 50% of the binding site was missing due to incomplete mitochondria sequence, binding site conservation was assumed. When considering all binding sites together, only 5 sequences were unconserved (1 *P. falciparum* (LR131448.1) and 4 *P. vivax* (JX444719.1, JX444725.1, AB550280.1, KY923385.1)).

Supplementary Tables

**Supplementary Table 1**. Summary of *in silico* assessment of binding site conservation for the Pan *Plasmodium* RPA-LF assay.

|  | | **RPA-LF Oligo** | | |
| --- | --- | --- | --- | --- |
| **Species** | **Binding Site State** | **FP** | **RP** | **Probe** |
| ***P. falciparum*** | Conserved | 428 | 428 (*222) | 427 |
|  | Unconserved | 0 | 0 | 1 |
| ***P. malariae*** | Conserved | 5 | 5 | 5 |
|  | Unconserved | 0 | 0 | 0 |
| ***P. ovale spp*** | Conserved | 3 | 3 | 3 |
|  | Unconserved | 0 | 0 | 0 |
| ***P. vivax*** | Conserved | 448 | 448 | 445 |
|  | Unconserved | 1 | 1 | 2 |
| ***P. knowlesi*** | Conserved | 5 | 5 | 5 |
|  | Unconserved | 0 | 0 | 0 |

(*) Assumed conservation due to incomplete sequencing.

**Supplementary Table 2.** Blastn screening output for the Pan Plasmodium RPA-LF assay oligos, utilising the nt database under default blastn conditions.

Please see the attachment Supplementary_Table_2.csv

**Supplementary Table 3**. Summary of assay outcomes when screening *P. falciparum* clinical samples, collected in 2019, from Ouélessébougou, Mali. Assay outcomes are marked as a Boolean for positive (1) and negative (0) detection, respectively.

| **Isolate** | **RPA Outcome** | **qPCR Outcome** | **qPCR Ct Value** |
| --- | --- | --- | --- |
| F62 | 1 | 1 | 26.29 |
| F4 | 1 | 1 | 24.67 |
| F25 | 1 | 1 | 24.75 |
| F1 | 1 | 1 | 26.09 |
| F34 | 1 | 1 | 23.87 |
| F74 | 1 | 1 | 21.44 |
| F53 | 1 | 1 | 26.51 |
| F32 | 1 | 1 | 20.74 |
| F68 | 1 | 1 | 22.59 |
| F84 | 1 | 1 | 25.96 |
| F30 | 1 | 1 | 23.50 |
| F9 | 1 | 1 | 24.25 |
| F24 | 1 | 1 | 22.95 |
| F48 | 1 | 1 | 19.26 |
| F66 | 1 | 1 | 21.19 |
| F22 | 1 | 1 | 24.54 |
| F57 | 1 | 1 | 26.68 |
| F59 | 1 | 1 | 23.75 |
| F45 | 1 | 1 | 21.65 |
| F28 | 1 | 1 | 24.37 |
| F86 | 1 | 1 | 28.84 |
| F98 | 1 | 1 | 25.26 |
| F90 | 1 | 1 | 25.10 |
| F92 | 1 | 1 | 25.48 |

**Supplementary Table 4.** Summary of assay outcomes when screening *P. vivax* clinical samples, collected between 2013-2015, from Acre, Brazil. Assay outcomes for detection are positive (1) or negative (0).

| **Isolate** | **RPA Outcome** | **qPCR Outcome** | **qPCR Ct Value** |
| --- | --- | --- | --- |
| V122 | 0 | 1 | 31.90 |
| V293 | 1 | 1 | 20.23 |
| V95 | 1 | 1 | 21.46 |
| V414 | 1 | 1 | 27.10 |
| V145 | 1 | 1 | 23.62 |
| V311 | 1 | 1 | 25.57 |
| V26 | 1 | 1 | 23.99 |
| V210 | 1 | 1 | 23.32 |
| V548 | 1 | 1 | 23.90 |
| V13 | 1 | 0 | NA |
| V227 | 1 | 1 | 22.94 |
| V556 | 1 | 1 | 14.95 |

**Supplementary Table 5.** Summary of assay outcomes when screening neglected *Plasmodium* clinical isolates, *P. ovale spp* (Po) and *P. malariae* (Pm), provided by the Malaria Reference Laboratory, (MRL). Assay outcomes for detection are positive (1) or negative (0).

| **Isolate** | **RPA Outcome** | **qPCR Outcome** | **qPCR Ct Value** |
| --- | --- | --- | --- |
| Pm MRL1 | 1 | 1 | 19.56 |
| Pm MRL2 | 1 | 1 | 19.52 |
| Pm MRL3 | 1 | 1 | 19.09 |
| Pm MRL4 | 1 | 1 | 18.83 |
| Pm MRL5 | 1 | 1 | 14.66 |
| Po MRL1 | 1 | 1 | 21.46 |
| Po MRL2 | 1 | 1 | 19.96 |
| Po MRL3 | 1 | 1 | 19.71 |
| Po MRL4 | 1 | 1 | 19.22 |
| Po MRL5 | 1 | 1 | 18.63 |

**Supplementary Table 6.** Screening of *Anopheles spp* collected across 2018-2019 from the Democratic Republic of Congo. Collection samples were speciated and the presence of a recent blood meal was determined. Assay outcomes for detection are positive (1) or negative (0).

| **Isolate** | ***Anopheles* Species** | **Blood Meal** | **Prior Screening** | **RPA Outcome** | **qPCR Outcome** | **qPCR Ct Value** |
| --- | --- | --- | --- | --- | --- | --- |
| 2A9 | *An. funestus* s.l | + | 0 | 0 | 0 | 0 |
| 2C4 | *An. funestus* s.l | + | 0 | 0 | 0 | 0 |
| 2D11 | *An. funestus* s.l | - | 0 | 0 | 0 | 0 |
| 2D7 | *An. funestus* s.l | + | 0 | 0 | 0 | 0 |
| 2G7 | *An. funestus* s.l | + | 0 | 0 | 0 | 0 |
| 3B4 | *An. gambiae* s.l. | - | 0 | 0 | 0 | 0 |
| 3B8 | *An. gambiae* s.l. | + | 1 | 1 | 1 | 25.45 |
| 3C4 | *An. gambiae* s.l. | - | 0 | 0 | 0 | 0 |
| 3C7 | *An. gambiae* s.l. | + | 0 | 0 | 0 | 0 |
| 3C8 | *An. gambiae* s.l. | + | 0 | 0 | 0 | 0 |
| 3D4 | *An. gambiae* s.l. | - | 0 | 0 | 0 | 0 |
| 3D8 | *An. gambiae* s.l. | + | 0 | 0 | 0 | 0 |
| 3E4 | *An. gambiae* s.l. | - | 1 | 1 | 1 | 27.23 |
| 3E7 | *An. gambiae* s.l. | + | 1 | 0 | 1 | 30.31 |
| 3E8 | *An. gambiae* s.l. | + | 0 | 0 | 0 | 0 |
| 3F4 | *An. gambiae* s.l. | - | 0 | 0 | 0 | 0 |
| 3F7 | *An. gambiae* s.l. | + | 0 | 0 | 0 | 0 |
| 3F8 | *An. gambiae* s.l. | - | 0 | 0 | 0 | 0 |
| 3G4 | *An. gambiae* s.l. | - | 0 | 0 | 0 | 0 |
| 3G7 | *An. gambiae* s.l. | + | 0 | 0 | 0 | 0 |
| 3G8 | *An. gambiae* s.l. | - | 0 | 0 | 0 | 0 |
| 3H4 | *An. gambiae* s.l. | - | 0 | 0 | 0 | 0 |
| 3H8 | *An. gambiae* s.l. | - | 0 | 0 | 0 | 0 |
| 4B2 | *An. gambiae* s.l. | + | 1 | 0 | 1 | 27.73 |
| 4B3 | *An. gambiae* s.l. | - | 0 | 0 | 0 | 0 |
| 4C2 | *An. gambiae* s.l. | + | 0 | 0 | 0 | 0 |
| 4C3 | *An. gambiae* s.l. | - | 0 | 0 | 0 | 0 |
| 4D2 | *An. gambiae* s.l. | - | 0 | 0 | 0 | 0 |
| 4D3 | *An. gambiae* s.l. | - | 0 | 0 | 0 | 0 |

**Supplementary Table 7.** Summary of assay outcomes when screening *An. stephensi* isolates and pools provided by the Malaria Transmission Facility, LSHTM. Assay outcomes for detection are positive (1) or negative (0).

| **Isolate** | **Blood Fed** | **Unfed** | **RPA Outcome** | **qPCR Outcome** | **qPCR Ct** |
| --- | --- | --- | --- | --- | --- |
| AC1 | 1 | - | 1 | 1 | 21.86 |
| AC2 | 1 | - | 1 | 1 | 22.98 |
| AC3 | 1 | - | 1 | 1 | 22.84 |
| AC4 | 1 | - | 1 | 1 | 22.8 |
| AC5 | 1 | - | 1 | 1 | 27.71 |
| AC6 | 1 | - | 1 | 1 | 27.6 |
| AC7 | 1 | - | 1 | 1 | 21.67 |
| AC8 | 1 | - | 1 | 1 | 25.49 |
| ACN1 | - | 1 | 0 | 0 | 0 |
| ACN2 | - | 1 | 0 | 0 | 0 |
| AP1 | 1 | 4 | 1 | 1 | 24.14 |
| AP2 | 1 | 4 | 1 | 1 | 23.72 |
| AP3 | 1 | 4 | 1 | 1 | 23.79 |
| AP4 | 1 | 4 | 1 | 1 | 24.31 |
| AP5 | 1 | 4 | 1 | 1 | 25.88 |
| AP6 | 1 | 4 | 1 | 1 | 24.12 |
| AP7 | 1 | 4 | 1 | 1 | 25.27 |
| AP8 | 1 | 4 | 1 | 1 | 26.36 |
| APN1 | - | 5 | 0 | 0 | 0 |
| APN2 | - | 5 | 0 | 0 | 0 |

Supplementary Figures


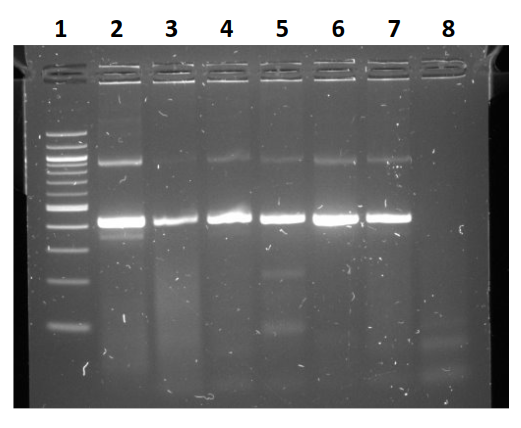


**Supplementary Figure 1**. Gel-electrophoresis of inclusivity samples, following incubation of the RPA-LF reaction at 95^o^C for 20 minutes to denature recombinase proteins: 1) NEB 100bp ladder, 2) *P. falciparum,* 3) *P. vivax,* 4) *P. malariae,* 5) *P. knowlesi,* 6) *P. ovale curtisi,* 7) *P. ovale wallikeri,* 8) Negative Control. Expected amplicon length of 409. Across all *Plasmodium spp*, a secondary amplicon was identified at ~900bp which is typical of RPA reactions which can form a ladder pattern upon the target amplicon forming secondary structures.


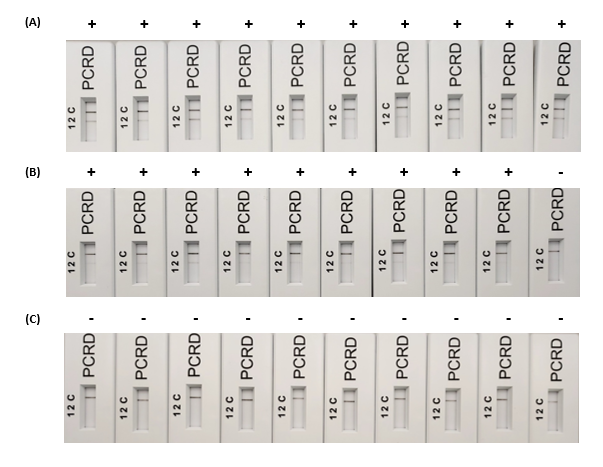


**Supplementary Figure 2**. Repeat screening of the Pan *Plasmodium* RPA-LF assay against *P. falciparum* serial dilutions 100 (A), 10 (B) and 1 (C) fg DNA/µl.


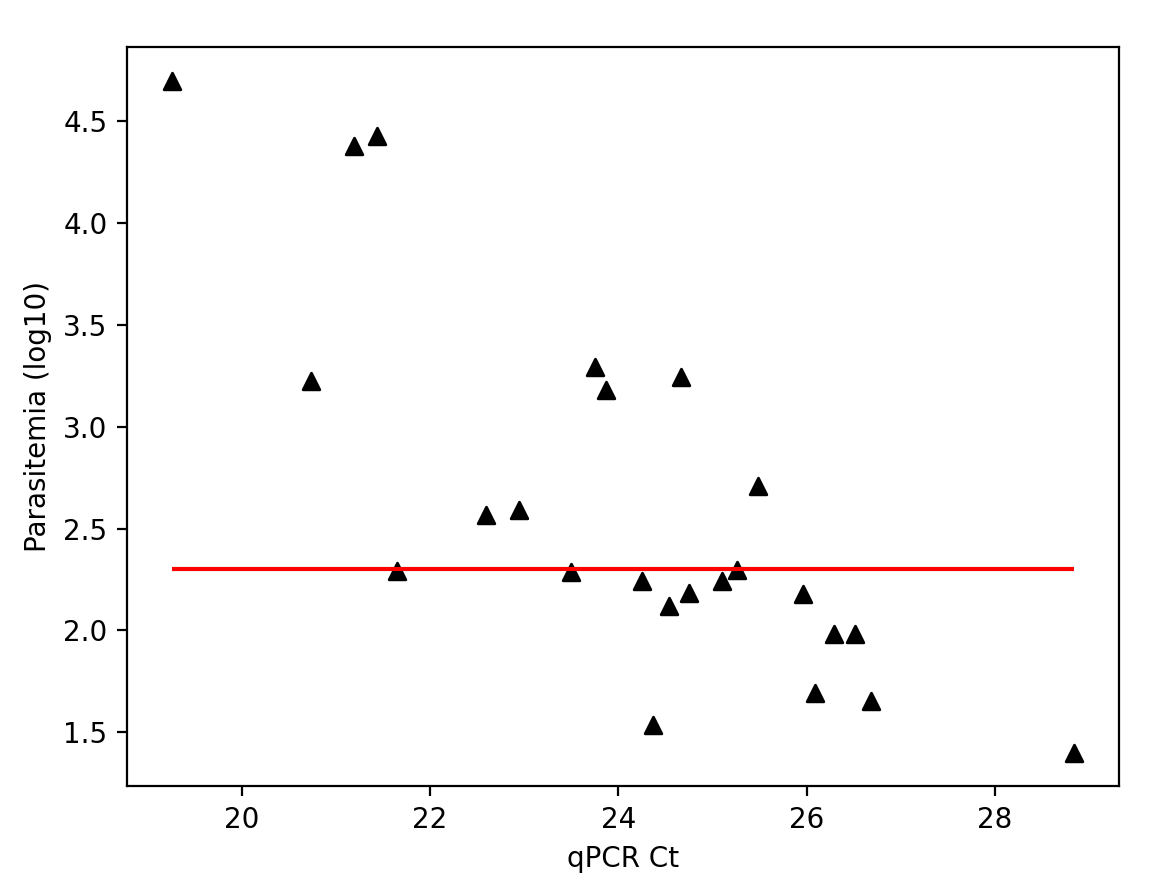

**Supplementary Figure 3**. *P. falciparum* clinical isolate and recorded parasitemia versus qPCR Ct value. As expected, isolates with a higher parasitemia have a lower Ct value due to the higher concentration of parasite DNA circulating. The red line indicates the 200 parasites/µl (0.004%) typically considered to be low density infections, according to the WHO Malaria RDT Performance report. In addition, a direct correlation between parasitemia and Ct value is not expected to be observed due to parasite DNA which may be circulating in the blood from lysed *Plasmodium spp* in addition to variability introduced during DNA extraction.


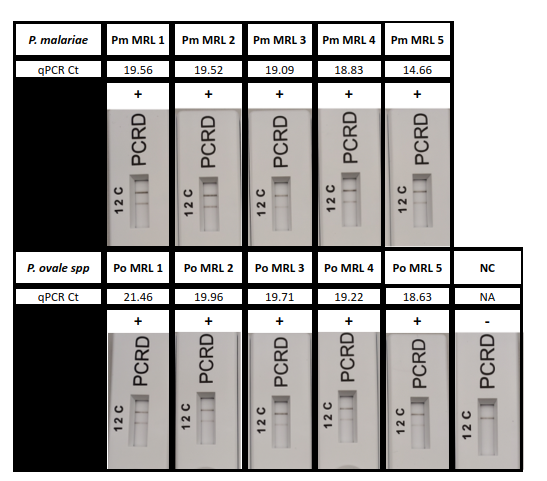


**Supplementary Figure 4.**  *P. malariae* and *P. ovale spp* clinical isolates screened using the Pan *Plasmodium* RPA assay.
